# Sero-epidemiology and associated factors of HIV, HBV, HCV and syphilis among blood donors in Ethiopia: a systematic review and meta-analysis

**DOI:** 10.1186/s12879-021-06505-w

**Published:** 2021-08-09

**Authors:** Mulugeta Melku, Sintayehu Ambachew, Bamlaku Enawgaw, Molla Abebe, Zegeye Abebe, Tekalign Deressa, Debasu Damtie, Belete Biadgo, Belay Tessema, Demeke Geremew, Asemarie Kebede, Berhanu Woldu, Takele Teklu, Elias Shiferaw

**Affiliations:** 1 Department of Hematology and Immunohematology, School of Biomedical and Laboratory Sciences, College of Medicine and Health Sciences, University of Gondar, Gondar, Ethiopia; 2 Department of Clinical Chemistry, School of Biomedical and Laboratory Sciences, College of Medicine and Health Sciences, University of Gondar, Gondar, Ethiopia; 3 Department of Human Nutrition, Institute of Public Health, College of Medicine and Health Sciences, University of Gondar, Gondar, Ethiopia; 4 Ethiopian Institute of Public Health, Addis Ababa, Ethiopia; 5 Department of Immunology and Molecular Biology, School of Biomedical and Laboratory Sciences, College of Medicine and Health Sciences, University of Gondar, Gondar, Ethiopia; 6 Department of Medical Microbiology, School of Biomedical and Laboratory Sciences, College of Medicine and Health Sciences, University of Gondar, Gondar, Ethiopia; 7 Department of Surgical Nursing, School of Nursing, College of Medicine and Health Sciences, University of Gondar, Gondar, Ethiopia

**Keywords:** Blood donors, Ethiopia, Meta-analysis, Sero-epidemiology, Systematic review, Transfusion-transmissible Infections

## Abstract

**Background:**

Transfusion transmissible infections (TTIs) remain a major public health problem in developing countries including Ethiopia. In Ethiopia, comprehensive information about sero-epidemiology of major TTIs is lacking at the national level. Therefore, this systematic review and meta-analysis was aimed at providing the pooled estimate of human immunodeficiency virus (HIV), hepatitis B virus (HBV), hepatitis C virus (HCV) and syphilis among blood donors in Ethiopia.

**Methods:**

Relevant studies published until May 31, 2019 were searched through PubMed/Medline, EMBASE, SCOPUS, HINARI, Cochrane database library, Web of Science, Google Scholar and Google. The methodological quality of articles was assessed using Joanna Brigg’s Institute critical appraisal checklist for prevalence and analytical studies. The pooled sero-epidemiology of HIV, HBV, HCV and syphilis were determined using the random-effects model. Heterogeneity between the studies was assessed using the I^2^ statistics. Publication bias was assessed by visual inspection of the funnel plot and Egger's statistics.

**Results:**

A total of 7921 articles were retrieved, and 7798 were screened for eligibility after duplicates removed. Forty-nine full-text articles were assessed for eligibility; of which 45 were eligible for qualitative and quantitative synthesis: categorized as 36, 34, 31 and 23 studies for estimations of HBV, HIV, HCV and syphilis, respectively. In the random-effects model, the pooled sero-epidemiology of HBV, HIV, HCV and syphilis was 5.20, 2.83, 0.93 and 1.50%, respectively. Moreover, being a male blood donor was significantly associated with HBV and syphilis infection, whereas being a replacement blood donor was significantly associated with a high burden of HIV, HBV and HCV infections.

**Conclusion:**

The pooled sero-epidemiology of major TTIs among blood donors was high. Therefore, there is a need to design prevention and control strategies in a comprehensive approach to reduce the burden.

**Supplementary Information:**

The online version contains supplementary material available at 10.1186/s12879-021-06505-w.

## Background

Blood is an invaluable, life-sustaining fluid. The loss of a large volume of blood due to casualties, hemorrhage, and other medical conditions lead to blood transfusion as part of the standard care to save a life. Because of this, blood transfusion is considered as an integral and essential element of a health care system [1, 2]. Though blood transfusion is one of the ways to save patients’ life in many cases, sometimes it poses the risk of TTIs. Supplying safe blood and preventing the transmission of infectious diseases are among the utmost important goals for blood transfusion organization [1]. Transfusion-transmissible infectious agents mainly HIV, HBV, HCV, and syphilis are among the greatest threats for the receivers if not rigorously screened [3].

According to the World Health Organization (WHO) 2016 report, the reactivity for markers of TTIs, outdated stock, and incomplete collection were the main reasons for the discard of donated blood. The median total discard rate was 10.9, 9.0, 6.7 and 5.7% in lower-middle-income, low-income, upper-middle-income, and high-income countries, respectively. Markers of TTIs are the most common reason for the discard, which accounted for a median discard rate of 7.4, 5.1, 3.9 and 1.1% in low-income, lower-middle-income, upper-middle-income, and high-income countries, respectively. Globally, it is estimated that 1.8 million donated blood were discarded due to TTIs reactivity in 2013 [4].

On another hand, according to the WHO report of 2020, 37.6 million people were living with HIV globally, with over two-thirds of who are living in the WHO African region. Some portion of HIV infection was through transfusion of blood and blood products. Similarly, reports from Sub-Saharan Africa showed that the median overall risk of becoming infected with HIV from a blood transfusion is 1 infection per 1000 units [5], and risk for 12.5% of post-transfusion viral hepatitis infection [6].

Viral hepatitis is the other global health burden. More than one million people die each year from HCV and HBV infections. According to the WHO 2017 report, chronic HBV infection affects over 257 million people worldwide. On the other hand, HCV is a leading cause of chronic liver disease, and persistent HCV infection is associated with cirrhosis, hepatocellular carcinoma, liver failure, and death [7]. Globally, the sero-prevalence of HCV ranges from 2 to 3%, with an estimated 71.1 million patients with active viremia, where 10.15 million are from sub-Saharan Africa [8]. Recent systematic review and meta-analysis showed that HCV was estimated to be 1.78% among blood donors in sub-Saharan Africa [9]. On the other hand, syphilis was also associated with blood donation, and its sero-prevalence among blood donors varies in Africa [10–13].

Implementing rigorous screening algorism of donated blood and blood products for markers of TTIs is the strategy to ensure the safety of blood donation. However, the risk of post-transfusion hepatitis and HIV is challenging in Sub-Saharan African countries; and patients who received blood transfusion develop post-transfusion hepatitis and HIV [5, 6]. Therefore, prevention and control of TTIs is the leading concern and priority agenda of WHO in Sub-Saharan Africa [14] and also the Ethiopian Federal Ministry of Health [15].

Systematic review and meta-analysis generates pooled and concrete evidence that would help to determine the distribution of the TTIs and contributes to planning the national strategies for containment and awareness-raising campaigns as well as to design specific preventive measures. As to the authors’ knowledge, limited systematic and meta-analysis were reported, each focusing on a single TTI type [16, 17]. Thus, systematically organized and strong evidence with regard to the sero-epidemiology and associated factors of the major TTIs among blood donors in Ethiopia is crucial. Therefore, the aim of this review was to estimate the epidemiology of TTIs among blood donors in Ethiopia by: (a) performing a systematic literature review, and (b) performing a meta-analysis on TTIs sero-prevalence and commonly reported risk factors of TTIs.

## Methods

### Context of the review

The context of this systematic review was limited to blood donors in Ethiopia. Ethiopia is one of the developing countries located in the sub-Saharan and horn of Africa having more 100 million people with an area of 1,100,000 km^2^.

### Design and protocol registration

The Preferred Reporting Items for Systematic Reviews and Meta-Analysis Protocols (PRISMA-P 2015 Guidelines) was used for this review [18]. The protocol had been registered in the PROSPERO with a registration number CRD42018076616.

### Eligibility criteria

Primary studies with full-text original articles written in English up to May 31, 2019 and having extractable data on sero-epidemiology of and/or factors associated with HIV, HBV, HCV and syphilis among blood donors in Ethiopia were included in the review. However, studies conducted among Ethiopian blood donors living abroad, repeated studies used the same dataset, and studies focused on the molecular Epidemiology of TTIs, case–control studies, case reports, case series and editorials were excluded from this review.

### Outcomes of the review

This review had two outcomes. The first outcome was to determine the pooled sero-epidemiology of HIV, HBV, HCV and syphilis among blood donors in Ethiopia. The secondary outcome was to identify factors associated with the sero-epidemiology HBV, HIV, HCV and syphilis.

### Search strategy

All relevant articles published were searched in PubMed/Medline, EMBASE, HINARI, SCOPUS, Cochrane database library, Google Scholar and Web of Sciences electronic databases. In addition, we searched grey literature in Goggle. The search terms were developed in accordance with the Medical Subject Headings Thesaurus (MeSH), and Boolean operators (AND, OR) were used for searching the articles. The following terms in combination with free text key terms; “transfusion transmissible infections”, “transfusion transmissible diseases”, “transfusion transmissible viral hepatitis”, “Transfusion transmitted infections”, “Transfusion transmitted viral hepatitis”, “HIV”, “Hepatitis B virus”, “TTIs” “Hepatitis C virus” “Syphilis”, “*T.pallidum*”, “blood donor” and “Ethiopia” were used. Also, hand searching of articles published in the Ethiopian Journal of Health Sciences, Ethiopian Medical Journal, Ethiopian Journal of Health and Development, and Ethiopian Journal of Health and Biomedical Sciences was conducted. Reference lists of relevant retrieved articles were used to identify any studies that were not retrieved through electronic database searching.

### Study selection and quality appraisal

First, retrieved articles were imported to EndNote X7 (Thomson Reuters, New York, USA), and duplicated articles were removed electronically, and manually if the citation style variation of different databases were noted. Then, articles were screened by their title and abstract independently by two groups comprising four authors each: group one (MM, ZA, MA, BB), and group two (SA, ES, BW, DG). Similarly, the two groups of authors had appraised the full-texts of the studies and in case of discrepancies, it was resolved by the third group of authors (BE, BT, TD, DD, TT, AK). In the case of articles eligible for full-text appraisal but not accessible in full text, the corresponding author communicated with the journal editorial office and/or publishers via email and/or phone. For assessment of the methodological quality of articles, the Joanna Brigg’s Institute critical appraisal tools for prevalence and analytical studies were used [19, 20].

### Data extraction

The data were extracted using Microsoft Excel format. Accordingly, the following pieces of information were collected: first author’s name, year of publication, year of data collection, length of the data collection period, sampling techniques (blood donors recruitment strategy), a diagnostic method used, total population participated, the number of blood donors who were sero-reactive for HIV, HBV, HCV and syphilis, blood donors type, median/mean age of the study participants, sex of study participants, and the number of male and female blood donors who were sero-reactive and non-reactive, sero-reactivity for each TTIs by donor type, and the reported odds ratio (OR) for the respective TTIs.

### Data analysis and interpretation

We used STATA version 14 software (Stata Corp LLC, Texas, USA) for analysis. The magnitude of heterogeneity between included studies was quantitatively measured by the index of heterogeneity (I^2^ statistics) [21]. Low, medium and high heterogeneities were noted where the I^2^ values are 25%, 50% and 75%, respectively. The significance of heterogeneity was determined by the P-value of I^2^ statistics and a P-value of < 0.05 was considered as evidence of heterogeneity. In the case of a medium and high level of heterogeneity between the included studies, Dersimonian and Laird random-effects model [22], sensitivity and subgroup analysis were used for estimating the pooled effect size. Considering the median year of sample collection as a factor, univariable random-effects meta-regression was performed to evaluate the trend of TTIs sero-prevalence over time.

Similarly, small-study effect or publication bias was evaluated using the visual funnel plot test, and Egger’s statistics. In the case of small-study effect, trim and fill analysis (Duval and Tweedie’s methods) was used to adjust the pooled estimates [23]. The ORs were extracted from the articles when reported, or calculated for each of the reported variables and pooled in a meta-analysis. Then OR with its 95% confidence interval was used to estimate the association between each TTIs and the reported factors. The results were presented using text and plots.

## Results

### Characteristics of included studies

A total of 7921 records were retrieved; of which 123 articles were removed due to duplication. During title/abstract screening, 7749 articles were also removed as their titles and abstracts were not relevant to the current review. One article eligible for full-text appraisal was excluded as the full-text article was not accessible after repeated contact of the corresponding author via email, and the journal editorial office [24]. The remaining 49 full-text articles were assessed for eligibility.

**Fig. 1:**
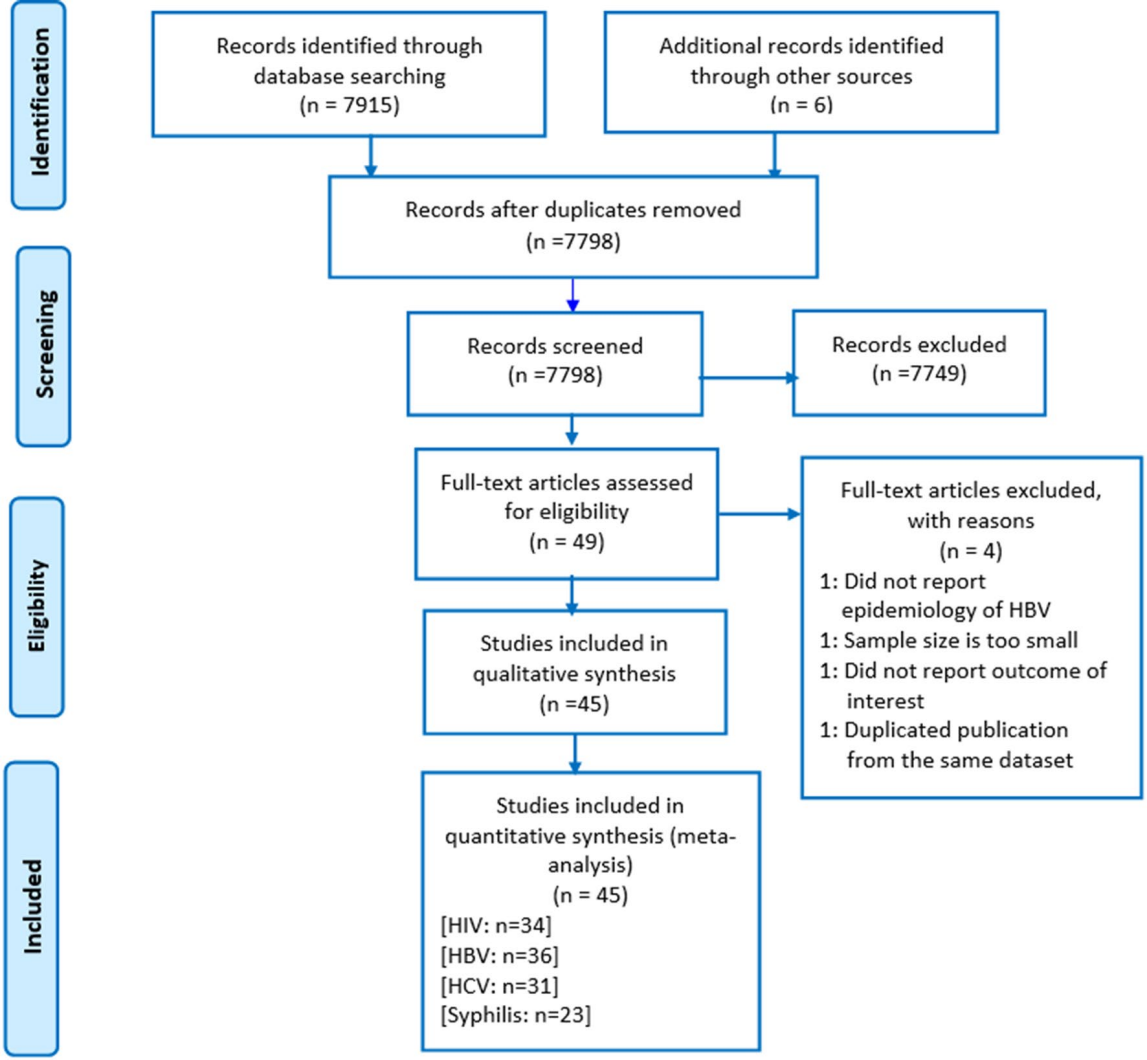
PRISMA flow diagram of studies reviewed, screened and included

Out of the total studies that underwent critical appraisal, 4 were excluded: 1 study did not report the epidemiology HBV infection [25]; 1 study the sample size is too small for epidemiological estimation [26]; 1 study did not clearly report the outcome interest [27], and the other study authored by Dessie et al. in 2008 [28] was a duplicate publication from the same dataset published in 2007 [29]. The remaining 45 studies were included in the qualitative and quantitative synthesis (Fig. 1).

Regarding the type of literature included in this review, 43 (95.6%) articles were published studies, and the rest were grey literature [30, 31], which involved a total 4,441,920 study participants. Twenty-four (53.3%) and 21 (46.7%) of the included studies were retrospective and prospective studies, respectively. Concerning the regions where these studies conducted, 17 (37.8%), 6 (13.3%), 6 (13.3%) and 5 (11.1%) of the studies were conducted in Amhara, Addis Ababa, South Nations and Nationalities, and Oromia regional state, respectively; whereas 4 (8.9%) of the studies were sub-national level studies.

Of all included studies, 36 reported HBV [3, 13, 29–62]; 34 reported HIV [3, 13, 29–33, 35–38, 42, 43, 45, 46, 48–58, 60–68]; 31 reported HCV [3, 13, 29–33, 35–38, 41–43, 45–51, 54, 55, 57, 58, 60–62, 69, 70]; and 23 reported syphilis [3, 13, 29–31, 33, 35, 36, 38, 42, 43, 45, 46, 49–52, 54, 57, 58, 60, 68, 71] prevalence among blood donors in Ethiopia. Regarding the distribution of each TTIs with sex and donor type of the study participants, it was found as follows: of 36 studies reported HBV, 24 and 9 reported sex and donor type, respectively; of 34 studies reported HIV, 24 and 8 studies reported sex and donor type, respectively; of 31 reported HCV, 19 and 7 reported sex and donor type, respectively; and of 23 studies reported syphilis, 14 and 5 studies reported sex and donor type, respectively (Table 1).

**Table 1:** Characteristics of studies included in the systematic review and meta-analysis of TTIs among blood donors in Ethiopia

First Author	Publication year	Sampling year	Study Design	Blood donor type	Setting	Sample Size	Blood donor by sex		HIV		HBV		HCV		Syphilis	
							Male	Female	HIV reactive	HIV Diagnosis method	HBVsAg reactive	HBVSAg Diagnosis method	Anti-HCV reactive	Anti-HCV Diagnosis Method	Syphilis Reactive	Syphilis Diagnosis Method
Asrie F et al.	2016	2015	Prospective	–	North Gondar	558	351	207	1.4%(8/558)	4th Gen ELISA	3.9% (22/558)	4th Gen ELISA	1.8% (10/558)	4th Gen ELISA	–	–
Teklemariam Z, et al.	2018	2008–2015	Retrospective	Both	Harar	11,382	9403	1978	0.6%(69/11382)	ELISA	4.4% (5001/11382)	ELISA	0.8% (91/11382)	ELISA	1.6% (182/11382)	ELISA
Habte Y et al.	2016	2009–2014	Retrospective	Both	Dire Diwa	4157	3434	723	–	–	3.73% (155/4157)	–	–	–	–	–
Sharew B et al.	2017	2008–2012	Retrospective	Both	Desie	9384	7514	1870	5.1%(476/9384)	4th Gen ELISA	–	–	–	–	–	–
Diressa T et al.	2018	2014–2017	Retrospective	Both	North Shewa	8460	5644	2816	0.25%(21/8460)	4th Gen ELISA	1.2% (103/8460)	4th Gen ELISA	0.32% (27/8460)	ELISA	0.71% (60/8460)	TPHA
Seyoum E, et al.	2005	2003	Prospective	–	Addis Ababa	408	–	–	4.2%(17/408)	–	–	–	–	–	–	–
Ambachew H, et al.	2018	2016	Prospective	–	Hawassa	2237	–	–	1.7%(38/2237)	4th Gen ELISA	4.7% (106/2237)	4th Gen ELISA	0.5% (11/2237)	ELISA	0.5% (11/2237)	ELISA
Degefa B et al.	2018	2011–2014	Retrospective	Both	Mekelle	10,728	3750	6978	1.03%(111/10728)	4th Gen ELISA	3.8% (407/10728)	4th Gen ELISA	1.33% (143/10728)	ELISA	–	–
Abate M et al.	2016	2010–2014	Retrospective	Both	Jigjiga	6827	6648	179	3.16%(216/6827)	4th Gen ELISA	9.48% (647/6827)	4th Gen ELISA	0.73% (50/6827)	ElISA	0.73% (50/6827)	RPR
Frommel D et al.	1993	1991	Prospective	Volunteer	Addis Ababa	220	133	87	–	2nd Gen ELISA	–	–	1.4% (3/220)	ELISA	–	–
Gelaw B et al.	2007	2002–2003	Prospective	-	Amhara & Tigrai	600	578	22	–	–	6.2% (37/600)	Immunochromatography	1.7% (10/600)	Immunochromatography	–	–
Gezahegn et al.	2012	2007–2010	Retrospective	Both	Jimma	3788	3034	754	–	ELISA	1.0% (39/3788)	–	–	–	–	–
Hundie GB et al.	2016	2013–2014	Prospective	Both	National	56,885	40,204	16,681	–	–	3.9% (2217/56885)	4th Gen ELISA	0.52% (294/56885)	–	–	–
Kebede W et al.	2017	2010–2015	Retrospective	Both	Jimma	10,733	7608	3125	0.2%(23/10733)	ELISA	3.05% (328/10733)	ELISA	0.2% (22/10733)	ELISA	0.14% (15/10733)	ELISA
Yemi A et al.	2011	2010	Retrospective	Both	Jimma	6,063	4802	1261	2.1%(129/6063)	ELISA	2.1% (126/6063)	ELISA	0.2% (10/6063)	ELISA	0.7% (44/6063)	RPR
Tsega E et al.	1995	1992–1994	Prospective	Volunteer	Addis Ababa	500	–	–	–	–	–	–	1.4% (7/500)	ELISA	–	–
Tsega E et al.	1987	1986	Prospective	Volunteer	Addis Ababa	459	409	50	–	–	11% (50/459	ELISA	–	–	–	–
Tessema B et al.	2010	2003–2007	Retrospective	Both	North Gondar	6361	5592	769	3.8%(239/6361)	ELISA	4.7% (298/6361)	ELISA	0.7% (35/6361)	ELISA	1.3% (83/6361)	RPR
Terefe JE et al.	Unpublished	2008–2013	Retrospective	Both	Addis Ababa	173,207	135,007	38,200	1.6%(2848/173207)	ELISA	5.0% (8743/173207)	ELISA	1.4% (2357/173207)	ELISA	0.1% (93/173207)	ELISA
Sisay Y et al.	1995	1987–1989	Retrospective	Both	National	19,408	16,735	2473	–	–	–	–	–	–	4.5% (870/19408)	–
Birhan W et al.	Unpublished	2016–2017	Prospective	Volunteer	Amhara	987	554	433	0.4%(4/987)	4th Gen ELISA	3.65% (36/987)	4th Gen ELISA	0.2% (2/987)	ELISA	0.91% (9/987)	RPR + TPHA
Ataro Z et al.	2018	2010–2013	Retrospective	Both	Dire Dawa	6376	5430	946	1.24%(79/6376)	ELISA	4.67% (275/6376)	ELISA	0.96% (61/6376)	ELISA	0.44% (28/6376)	RPR
Mohammed Y et al.	2016	2010–2013	Retrospective	Both	Jijiga	4224	4171	53	0.1% (6/4224)	ELISA	10.9% (460/4224)	ELISA	0.4% (17/4224)	ELISA	0.1% (4/4224)	ELISA
Assefa A et al.	2013	2007–2008	Prospective	Both	Bahir Dar	2384	2177	207	–	–	4.11% (98/2384)	ELISA	0.63% (5/2384)	ELISA	–	–
Biadgo B et al.	2017	2010–2012	Retrospective	Both	North Gondar	6741	5311	1160	2.24% (145/6471)	4th Gen ELISA	3.6% (233/6471)	3rd Gen ELISA	0.8% (51/6471)	3rd Gen ELISA	–	–
Berhanselase M	2016	2009–2103	Retrospective	Both	Hawassa	6306	5471	835	1.6% (101/6306)	4th Gen ELISA	4.8% (303/6306)	ELISA	0.6% (38/6306)	ELISA	0.5% (32/6306)	RPR
Biseteg FS et al.	2016	2015	Prospective	-	Wolaytsodo	390	291	99	6.4% (25/390)	4th Gen ELISA	9.5% (37/390)	3rd Gen ELISA	8.5% (33/390)	3rd Gen ELISA	7.9% (31/390)	ELISA
Bonja F et al.	2017	2015	Prospective	Both	Hawassa	384	258	126	1.6% (6/384)	–	4.2% (16/384)	–	0.5% (2/384)	–	0.8% (3/384)	
Kassu A et al.	2006	1995–2002	Retrospective	Both	North Gondar	11,204	10,659	545	9.9% (1109/11204)	ELISA	–	–	–	–	–	–
Rahlenbeck SL et al.	1997	1994–1995	Prospective	Both	North Gondar	2186	2186	0	14.5% (317/2186)	ELISA	14.4% (79/549)	3rd Gen latex test	–	–	12.9% (283/2186)	RPR
Zewde D et al.	1991	1989	Prospective	-	Addis Ababa	408	–	–	2.9% (12/408)	ELISA	10.8% (44/408)	ELISA	–	–	–	–
Desie A et al.	2007	2006	Prospective	Both	Bahir Dar	324	283	41	11.7% (38/324)	4th Gen ELISA	25% (81/324)	3rd Gen ELISA	13.3% (43/324)	3rd Gen ELISA	1.2% (4/324)	RPR
Ramos JM et al.	2008	1998–2006	Retrospective	Both	west Arsi	3305	2754	551	1.5% (51/3305)	Immunochromatography	–	–	–	–	–	–
Ramos JM et al.	2016	2007–2012	Retrospective	–	west Arsi	2606	–	–	0.73% (19/2606)	Immunochromatography	4.95% (129/2606)	Immunochromatography	1.68% (44/2606)	Immunochromatography	0.84% (22/2606)	RPR
sentlens RE et al.	2002	1991	Prospective	–	Ethiopia	2610	2069	505	7.7% (207/2610)	ELISA	–	–	–	–	–	–
Dro E et al.	2008	2004	Prospective	–	North Gondar	600	537	63	4.5% (27/600)	ELISA	8.2% (49/600)	ELISA	5.8% (35/600)	ELISA	–	–
Kabato AA et al.	2016	2015	Prospective	–	Arba Minch	359	179	162	–	–	4.7% (17/359)	ELISA	–	–	–	–
Shiferaw E et al.	2019	2014/2015- 2017/2018	Retrospective	Both	Bahir Dar	35,435	23,032	12,403	0.5% (160/35435)	ELISA	3.9% (1389/35435)	ELISA	0.6% (203/35435)	ELISA	1.2% (429/35435)	ELISA
Azerefegn E et al.	2018	2015	Retrospective	Both	Hawassa	6849	–	–	–	4th Gen ELISA	–	–	–	–	–	–
Berhanu S et al.	2018	2017	Retrospective	Both	Debre tabor	7255	–	–	–	4th Gen ELISA	–	–	–	–	–	–
Bialfew Y et al.	2018	2018	Prospective	Volunteer	Debre Markos	403	–	–	–	ELISA	–	–	–	–	–	–
Heyredin I et al.	2019	2018	Prospective	Both	Dire Dawa	500	–	–	–	ELISA	–	–	–	–	–	–
Assefa A et al.	1994	1989–1993	Retrospective	–	Gondar	3696	3066	630	10.85 (401/3696)	ELISA	–	–	–	–	7.9% (181/2299)	RPR
Negash M et al.	2019	2018	Prospective	Volunteer	Debre Tabor	310	198	112	2.58% (8/310)	ELISA	5.81% (18/310)	ELISA	4.2%(13/310)	ELISA	–	–
Tigabu et l	2019	2018	Retrospective	Volunteer	Gondar	5983	5118	856	2.5%(147/5983)	4th gen ELISA	4.1%(244/5983)	3rd gen ELISA	1.6%(98/5983)	3rd gen ELISA	–	–

*ELISA* enzyme linked Immunosorbent Assay, *gen* generation, – represents that the given characteristic is not reported, *HBV* hepatitis B virus, *HBSAg* hepatitis B surface antigen, *HCV* hepatitis C virus, *HIV* human immunodeficiency virus, *RPR* rapid plasma regain, *TPHA* treponema pallidum hemagglutination assay, *TTIs* transfusion transmissible infections, *Both* represents the blood donor types are volunteer and replacement type

### Pooled estimates of TTIs

#### The Pooled estimate and trend of HIV

**Fig. 2:**
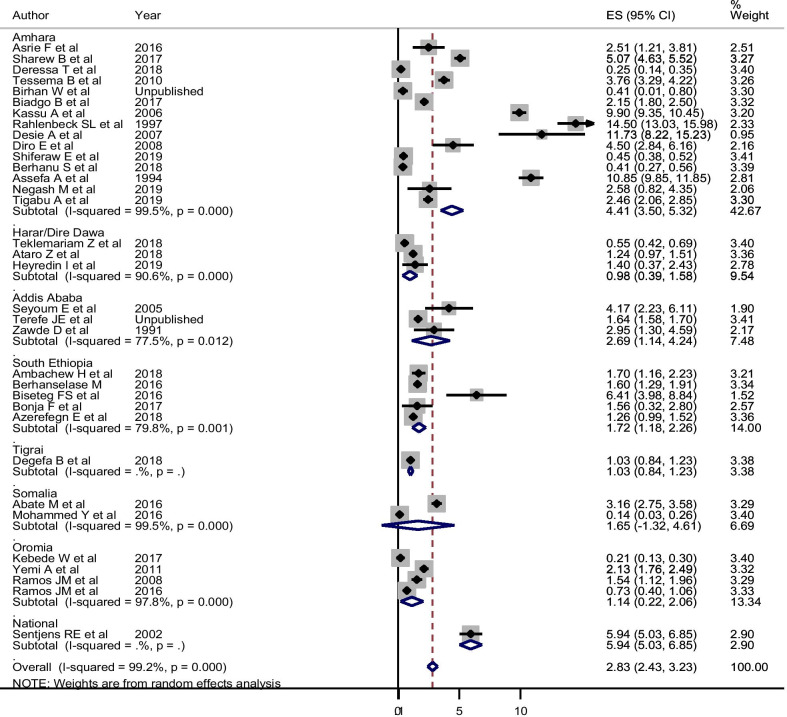
Pooled estimate of HIV using random-effect model among blood donors in Ethiopia: a subgroup analysis by region. *ES* estimated Prevalence of HIV

For the estimation of the pooled prevalence of HIV among blood donors, 34 articles with a total of 355, 026 participants’ data were used. These studies reported varying prevalence, ranging from 0.14% to 14.5%. In the random-effect model, the pooled size effect of HIV was 2.83% (95% CI 2.43, 3.23%) (Fig. 2). The trend of HIV showed that the seroprevalence showed a decrement from 1989 to 2018. The random-effects meta-regression analysis also confirmed that the pooled seroprevalence of HIV was negatively but significantly associated with the midpoint year of the data collection period (Coefficient = − 0.089; 95% CI − 0.133, − 0.045; *P* < 0.001). The seroprevalence estimate of HIV changed from 8.84% (95% CI 5.96, 17.73%) before 2000 to 1.20% (95% CI 0.87, 1.53%) during 2015–2018 (Table 2).

**Table 2:** Trends of the pooled sero-epidemiology of TTIs among blood donors in Ethiopia (1986–2018)

The trend of pooled sero-prevalence of HIV among blood donors in Ethiopia (1989–2018)								
Study year (based on mid-term year)	Number of included studies	Total No. of HIV sero-reactive case	Total No. of Population	Pooled prevalence of HIV %(95% CI)	Heterogeneity		Meta-regression (effect of year on HIV prevalence)	
					I^2^ %	P-value	Coefficient(95% CI)	P-value
Before 2000	5	1994	20,103	8.84 (5.96, 17.73)	97.7	< 0.001	− 0.089 (− 0.133, − 0.045)	< 0.001
2000–2004	3	95	4313	3.27 (1.02, 5.53)	88.5	< 0.001		
2005–2009	3	296	9291	4.55 (1.67, 7.44)	98.6	< 0.001		
2010–2014	11	4197	251,971	1.70 (1.14, 2.26)	99.3	< 0.001		
2015–2018	12	546	69,348	1.20 (0.87, 1.53)	94.9	< 0.001		
Total	34	7128	355,026	2.83 (2.43, 3.23)	99.2	< 0.001		

The trend of pooled sero-prevalence of HBV among blood donors in Ethiopia (1986–2018)								
Study year (based on mid-term year	Number of included studies	Total No. of HBV sero-reactive case	Total No. of Population	Pooled prevalence of HBV % (95% CI)	Heterogeneity		Meta-regression (effect of year on HBV prevalence)	
					I^2^ %	P-value	Coefficient (95% CI)	P-value
Before 2000	3	173	1415	12.03 (9.72, 14.34)	46.2	0.156	− 0.029 (− 0.078, 0.02)	0.25
2000–2004	2	86	1200	7.10 (5.14, 9.05)	44.6	0.179		
2005–2009	5	645	14,985	6.72 (4.03, 9.40)	98.7	< 0.001		
2010–2014	13	14,652	310,478	4.76 (4.05, 5.47)	98.6	< 0.001		
2015–2018	13	2240	63,261	4.30 (3.35, 5.24)	96.8	< 0.001		
Total	36	17,796	391,339	5.20 (4.64, 5.77)	98.4	< 0.001		

The trend of pooled sero-prevalence of HIV among blood donors in Ethiopia (1991–2018)								
Study year (based on mid-term year	Number of included studies	Total No. of HCV sero-reactive case	Total No. of Population	Pooled prevalence of HCV % (95% CI)	Heterogeneity		Meta-regression (effect of year on HCV prevalence)	
					I^2^ %	P-value	Coefficient (95% CI)	P-value
Before 2000	2	10	720	1.40 (0.54, 2.26)	0.0	1	− 0.041 (− 0.093, 0.01)	0.114
2000–2004	2	45	1200	3.69 (− 0.36, 7.74)	93.0	< 0.001		
2005–2009	4	137	10,136	1.86 (0.81, 2.90)	95.2	< 0.001		
2010–2014	12	3182	306,321	0.71 (0.41, 1.00)	98.7	< 0.001		
2015–2018	11	453	62,499	0.83 (0.55, 1.11)	91.2	< 0.001		
Total	31	3827	380,876	0.93 (0.73, 1.13)	97.3	< 0.001		

The trend of pooled sero-prevalence of Syphilis among blood donors in Ethiopia (1989–2018)								
Study year (based on mid-term year	Number of included studies	Total No. of Syphilis sero-reactive case	Total No. of Population	Pooled prevalence of Syphilis % (95% CI)	Heterogeneity		Meta-regression (effect of year on Syphilis prevalence)	
					I^2^	P-value	Coefficient (95% CI)	P-value
Before 2000	3	1334	23,893	8.39 (3.71, 13.08)	98.8%	< 0.001	− 0.085 (− 0.156, − 0.015)	0.021
2000–2004	–	–	–	–	–	–		
2005–2009	4	113	13,446	0.83 (0.06, 1.59)	96.2%	< 0.001		
2010–2014	8	413	227,743	0.50 (0.29, 0.71)	97.3%	< 0.001		
2015–2018	8	614	55,648	1.04 (0.67, 1.40)	90.8%	< 0.001		
Total	23	2474	320,730	1.50 (1.20, 1.80)	99.0%	< 0.001		

– represents that the given characteristic is not reported, *CI* confidence interval, *HBV* hepatitis B virus, *HCV* hepatitis C virus, *HIV* human immunodeficiency virus, *TTIs* transfusion transmissible infections

The influential analysis revealed that no single study was outside the 95% CI of the pooled estimate indicating that omitting each study did not affect the pooled estimate. Regarding the publication bias, the visual funnel plot seems symmetric, suggesting that there was no small-study effect. The objective-based test, egger’s statistics, confirmed that there was no small-study effect (Coefficient of bias = 3.69, P = 0.111). Since the degree of heterogeneity between the included studies was higher, subgroup analysis was done considering the “region” as a grouping variable. In the random-effects model, the pooled estimated prevalence of HIV was 4.41% (95% CI 3.50, 5.32%) in Amhara region, 1.72% (95% CI 1.18, 2.26%) in South Nations and Nationalities, 1.14% (95% CI 0.22, 2.06%) in Oromia, and 0.98% (95% CI 0.39, 1.58%) in Harar/Dire Dawa (Fig. 2).

##### HIV and its association with sex and donor type

**Fig. 3:**
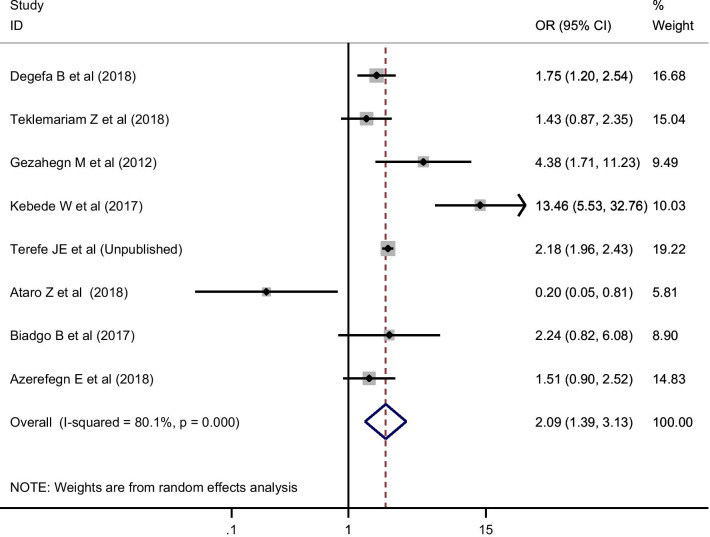
Effect size of donor type on pooled estimate of HIV using random-effect model

Factors such as sex and donor type were assessed to estimate their effect size on the pooled estimate of HIV. Twenty four and 8 studies had reported the distribution of HIV by sex and donor type, respectively. In the random-effects model, being a replacement blood donor was more likely to have HIV infection among blood donors in Ethiopia [Odds ratio (OR)] = 2.09; 95% CI 1.39, 3.13; I-squared = 80.1%) (Fig. 3). However, sex had no association with HIV infection (OR = 1.09; 95% CI 0.85, 1.40; I-squared = 86.1%). In both cases, there was no publication bias as evidenced by egger’s statistics (P > 0.05) and the visual funnel plot.

#### The Pooled estimate and trend of HBV

**Fig. 4:**
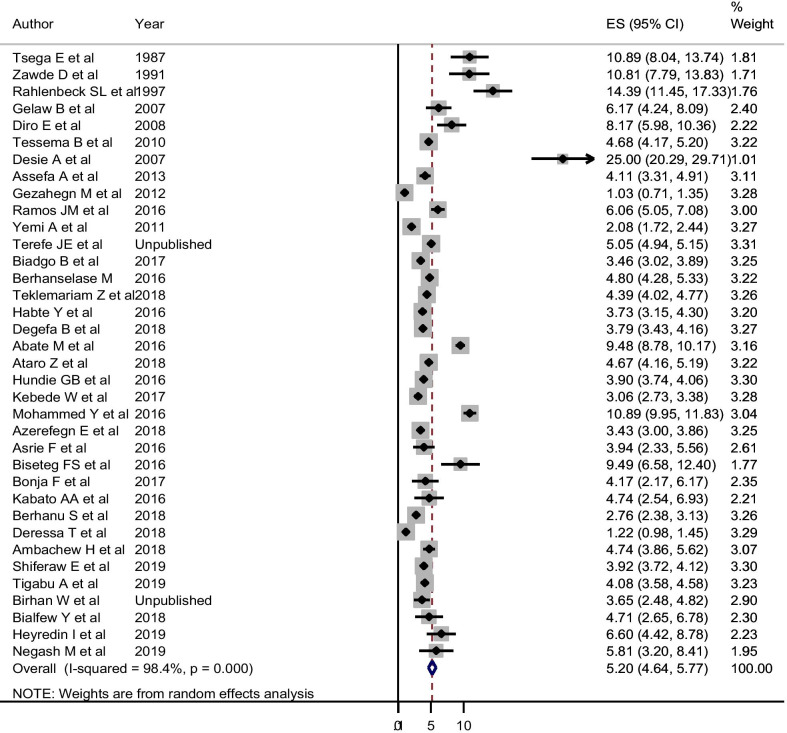
Pooled sero-epidemiology of HBV among blood donor in Ethiopia, Random-effect model. *ES* estimated Prevalence of HBV

Regarding HBV among blood donors in Ethiopia, 36 articles which include 391, 339 study participants were used for estimation. These studies reported varying prevalence, ranging from 1.03 to 25%. In the random-effects model, the pooled seroprevalence of HBV was 5.20% (95% CI 4.64, 5.77%; with I-squared = 98.4%). The trend analysis also showed that a decrement from 12.03% (95% CI 9.72, 14.34%) before 2000 to 4.30% (95% CI 3.35, 5.24%) during 2015–2018. Though the trend seems to decline from 1986 to 2018, the random-effects meta-regression analysis showed that the decline is not significantly associated with the midpoint year of the data collection period (Coefficient = − 0.029; 95% CI − 0.078, 0.02, P = 0.25) (Table 2). The subgroup analysis revealed that there were considerable variations of HBV estimate across regions in Ethiopia. The visual funnel plot was symmetric, suggesting no publication bias. Likewise, the egger’s statistics showed that there was no small-study effect on the pooled estimate of HBV infection (P = 0.450, coefficient of bias = 1.42) (Fig. 4).

##### HBV and its association with sex and donor type

**Fig. 5:**
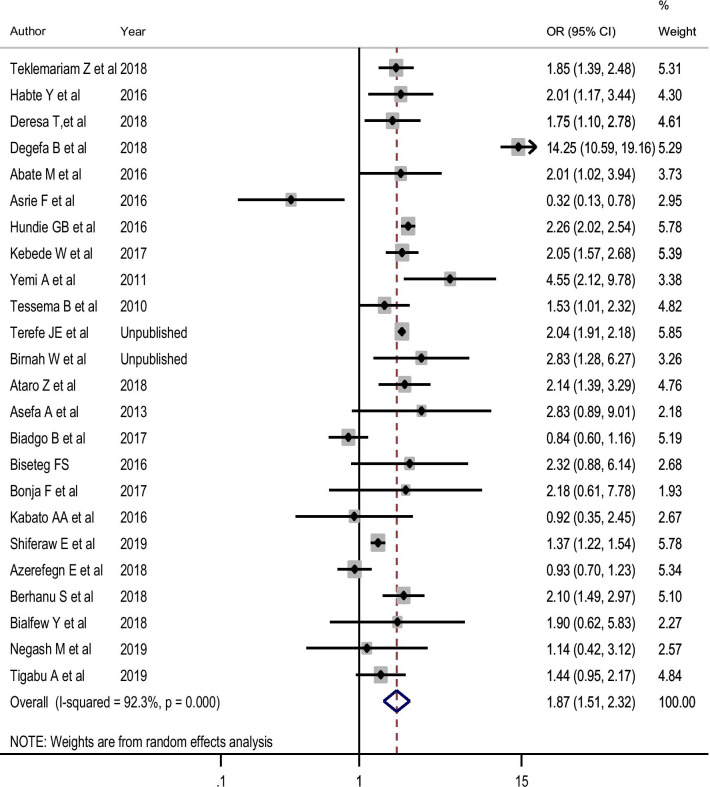
Effect size of sex on pooled estimate of HBV using random-effect model

**Fig. 6:**
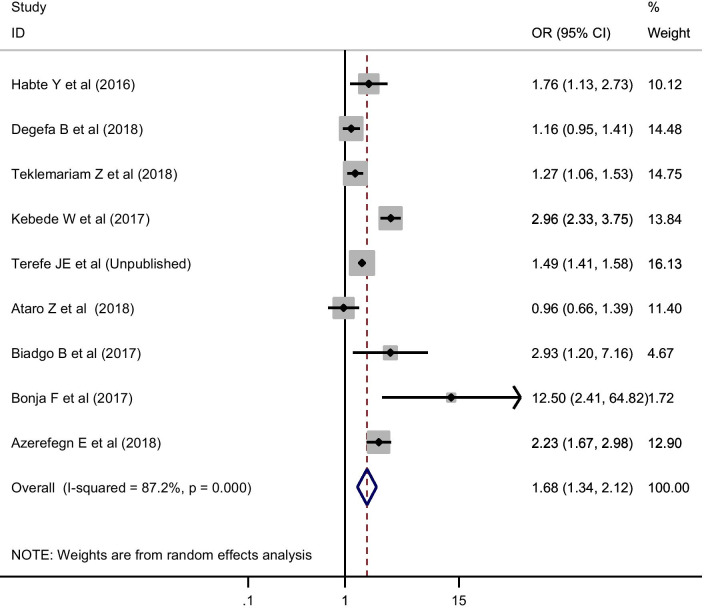
Effect size of donor type on pooled estimate of HBV using random-effect model

As there was high-level heterogeneity between the studies included for the estimation of the effect size of sex and donor type on HBV, a random-effects model was fitted. Therefore, male blood donors (OR = 1.87; 95% CI 1.51, 2.32) (Fig. 5), and replacement blood donors (OR = 1.68; 95% CI 1.34, 2.12) (Fig. 6) were more likely to be HBV sero-reactive. Besides, the assessment of publication bias revealed that there was no evidence of a small-study effect in both factors (P > 0.05).

#### The Pooled estimate and trend of HCV

**Fig. 7:**
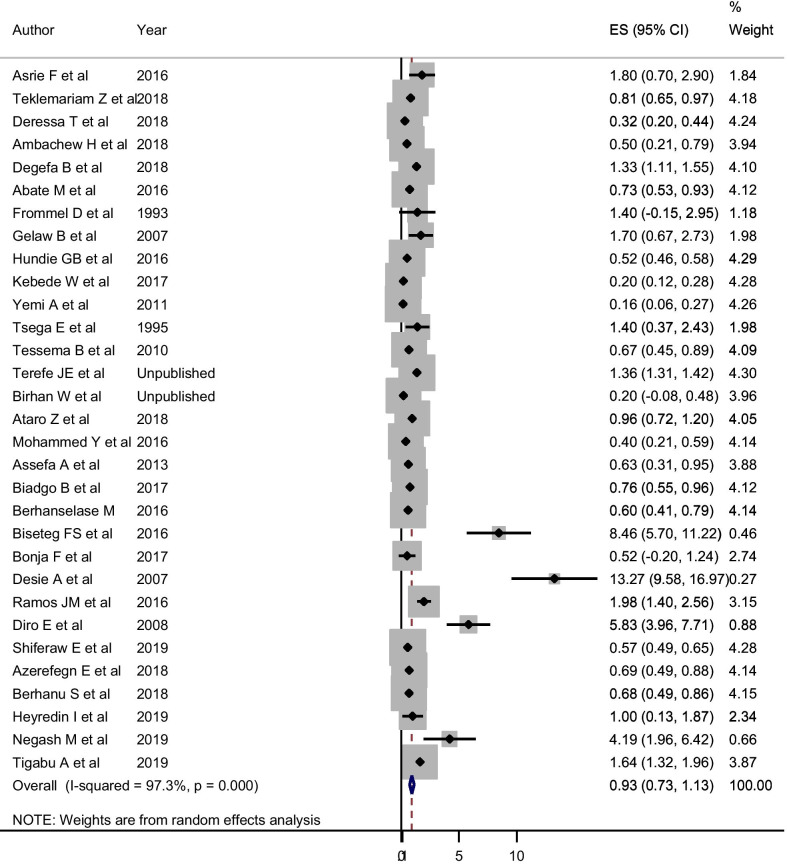
Pooled estimate of HCV among blood donors in Ethiopia in random-effect model. *ES* estimated Prevalence of HCV

**Fig. 8:**
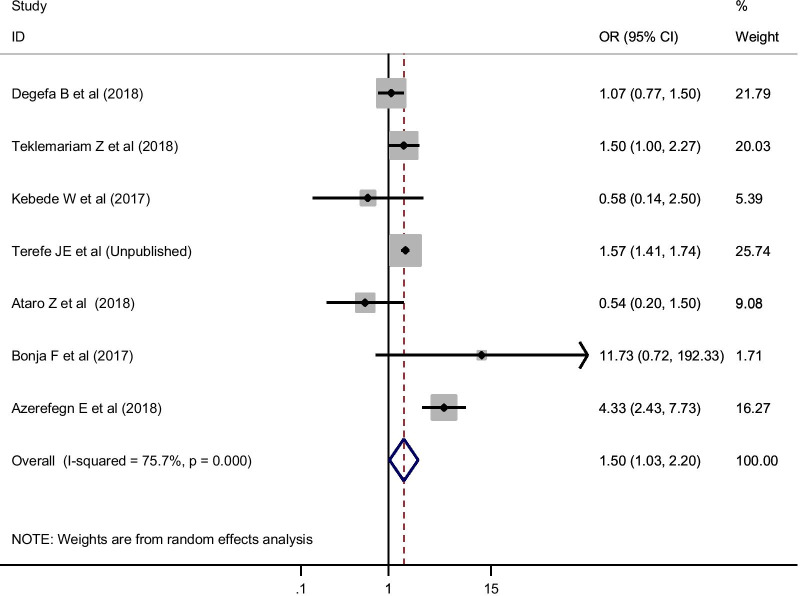
Effect size of donor type on pooled estimate of HCV using random-effect model

For the estimation of HCV among blood donors, 31 studies with a total of 380, 876 study participants were included. These studies reported varying prevalence, ranging from 0.2% to 13.3%. In the random-effects model, the pooled seroprevalence of HCV was 0.93% (95% CI 0.73, 1.13) (Fig. 7). In the random-effects meta-regression analysis, the pooled estimate of HCV showed a decrement from 1991 to 2018 but not statistically significant (Coefficient = − 0.041; 95% CI − 0.093, 0.01; *P* = 0.114). The pooled estimate changed from 1.40% (95% CI 0.54, 2.26%) before 2000 to 0.83% (95% CI 0.55, 1.11%) during 2015–2018 (Table 2). Egger’s statistics indicated that there was no small-study effect (coefficient of bias = 1.24; P = 0.446). About the effect size of sex and donor type on the pooled estimate of HCV, being a replacement blood donor was significantly associated with HCV (OR = 1.50; 95% CI 1.03, 2.20; I-squared = 75.7%) (Fig. 8). However, sex was not associated with HCV (OR = 1.24; 95% CI 0.95, 1.51). In both factors, there was no evidence of a small-study effect (P > 0.05).

#### The Pooled estimate and trend of Syphilis

**Fig. 9:**
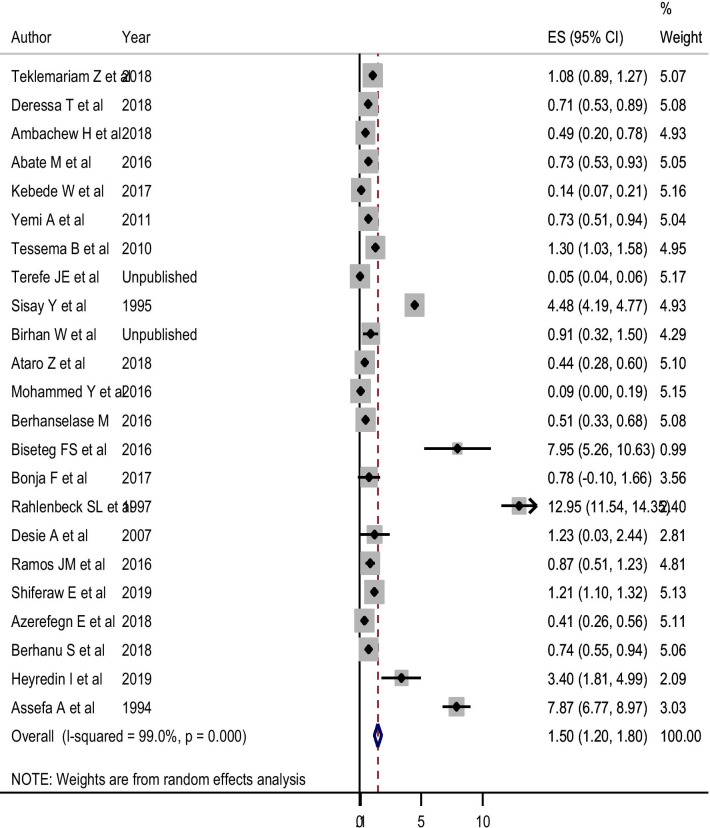
Pooled Estimate of Syphilis Sero-epidemiology among Blood donors in Ethiopia. *ES* estimated Prevalence of Syphilis

With regard to syphilis, 23 studies comprising 32, 0730 study participants were eligible for estimating its pooled sero-epidemiology. These studies reported varying prevalence, ranging from 0.1% to 12.9%. In the random-effects model, the pooled estimate of syphilis was 1.50% (95% CI 1.20, 1.80%) (Fig. 9). The pooled estimate declined from 8.39% (95% CI 3.71, 13.08%) before 2000 to 1.04% (95% CI 0.67, 1.40%) during 2015–2018. The trend analysis showed a decrement from 1989 to 2018. This has been supported by the random-effects meta-regression analysis, and the analysis confirmed that the pooled seroprevalence of Syphilis was negatively but significantly associated with the midpoint year of the data collection period (Coefficient = − 0.085; 95% CI − 0.156, − 0.015; *P* = 0.021) (Table 2).

As there was a publication bias evidenced by egger’s statistics (P < 0.001), Duval and Tweedie’s trim and fill analysis was done to adjust the final pooled estimate of syphilis sero-epidemiology. For the adjustment of an estimate, 12 studies were filled, and it was found that the pooled estimated prevalence in the random-effects model was found to be 0.11% (95% CI 0.061, 0.19%).

**Fig. 10:**
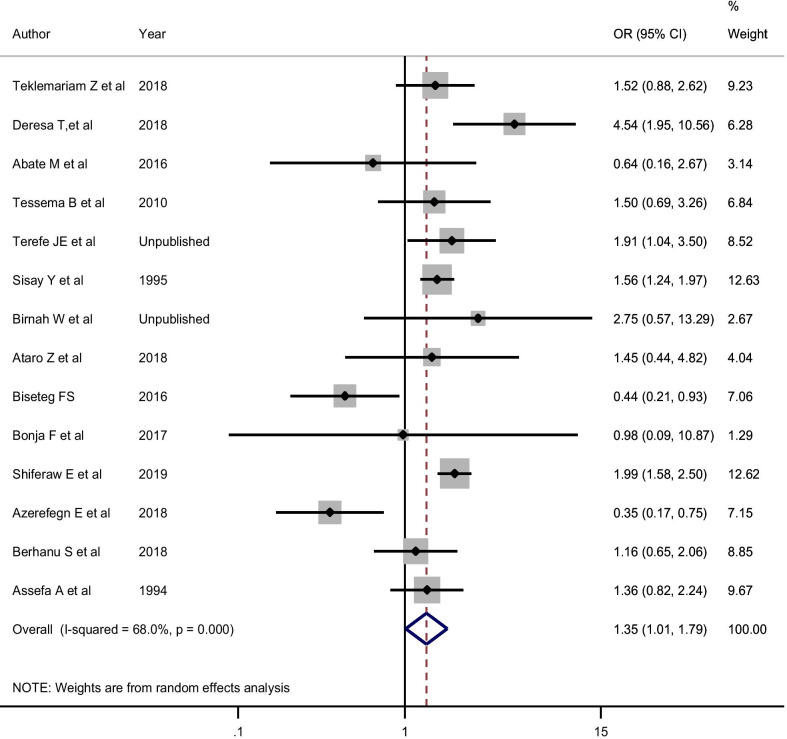
The effect size of sex on pooled estimate of syphilis using random-effect model

Concerning the effect size of sex and donor type on the sero-epidemiology of syphilis, being a male blood donor was more likely to be sero-reactive for syphilis compared to being a female donor in the random-effects model (OR = 1.35; 95% CI 1.01, 1.79**;** I-squared = 68%) (Fig. 10). Unlike sex, donor type was not associated with syphilis sero-reactivity (OR = 1.84; 95% CI 0.56, 6.07; I-squared = 88.4%). No publication bias was observed for both factors (P > 0.05).

## Discussion

Blood donation is becoming one of the lifesaving emergency services in medical and obstetrics services. Despite its advantage in lifesaving practice, blood transfusion is a potential risk for transmission of TTIs, such as HBV, HIV, HCV and syphilis [72]. In this review, the pooled sero-epidemiology of HBV, HIV, HCV and syphilis among blood donors in Ethiopia was 5.20% (95% CI 4.64, 5.77%), 2.83% (95% CI 2.43, 3.23%), 0.93% (95% CI 0.73, 1.13%) and 1.50% (95% CI; 1.20, 1.80%), respectively.

The pooled sero-epidemiology of HBV was higher than the estimates of a systematic review conducted in Iran (0.7%) [73], Eastern Mediterranean and Middle Eastern Countries (2.03%) [74] and European countries (0.74%) [75]. The possible reasons for the observed discrepancy might be due to the differences in cultural practices with regard to prevention of TTIs, and economic status which results in variation in vulnerability for sexually transmitted diseases. In addition, variation in the time period, the strength of preliminary screening of donors and factors related to testing algorithms used for screening might also be the possible reason for the discrepancy in the sero-epidemiology of HBV.

Moreover, our review showed that male blood donors (OR = 1.87; 95% CI 1.51, 2.32) were more likely to be HBV sero-reactive than females. This was in line with previously established studies in south Iran [76], Philippines [77], Ghana [78] and Egypt [79]. Another study also reported that females were less likely to be positive for HBV [80]. Almost 95% of HBV infected persons clear HBV by developing a protective Hepatitis B Surface Antibody (HBsAb) when HBV infection is acquired during adulthood, and only 5 to 10% of HBV infected persons will develop a persistent infection. Particularly, due to the slower plasma extinction rate in males, males are about 1.5 times more likely to develop chronic HBV infection relative to females, suggesting that sex is one of the genetic determinants that affect disease outcome [81]. On the other hand, replacement blood donors (OR = 1.68; 95% CI 1.34, 2.12) were more likely to be HBV sero-reactive compared to volunteer blood donors. This result was supported by earlier studies in which the reported prevalence of HBV among replacement donors was higher than that of voluntary donors [82, 83]. This could be the fact that during replacement donation, the donor might not consider the details of all his/her previous risky behaviours for TTIs as compared to volunteer donors.

Similarly, the pooled sero-epidemiology of HIV was higher than the estimate done in Iranian blood donors (0.0079%) [1]. The possible reasons for this difference might be attributable to the type of donors’ recruited (volunteer versus replacement), and the testing algorithms and kits used to diagnose HIV. In addition, the discrepancy was explained by the variation of the study period in which studies included for the estimation, as the Iranian included studies published since 1996, whereas our estimate included studies published since 1989.

Regarding factors associated with HIV, our review showed that being a replacement blood donor was more likely to have HIV infection compared to volunteer donors (OR = 2.09; 95% CI 1.39, 3.13). A consistent result had been reported previously in different countries [82, 83]. This might be partly explained by that, during replacement blood donation, family members of blood donors may not pass through strict preliminary screening as a result of the emergency nature of the case. Nevertheless, the prevalence of HIV was not significantly associated with sex. But other studies reported that the prevalence was high among males than females in the South of Iran [1, 76]. On the other hand, a study conducted in Iran in 2017 revealed that the seroprevalence of HIV among females was high [84]. This discrepancy in HIV report in terms of gender could be due to differences in the age status of employed donors and risky personal behaviours.

The pooled sero-epidemiology of HCV was 0.93% (95% CI 0.73, 1.13%). This estimate was consistent with estimates of first-time blood donors from European countries (0.02–3.3%). Our estimate is lower than an estimated prevalence of HCV Chinese blood donors (8.6%) [85]. The plausible reasons for the difference might be due to; firstly, the variation in the method of diagnosis, for example, the systematic review done in China included studies that used molecular techniques; secondly, the difference in population phenomena between countries like population migration, and thirdly, the difference in practices related to safe sexual practice. In addition, one of the major reasons for the variation in HCV prevalence is probably related to different safety regulations and variations in the level to which they are reinforced across countries (Additional file 1: Table S1, Additional file 2: Figure S1, Additional file 3: Figure S2, Additional file 4: Figure S3, Additional file 5: Figure S4, Additional file 6: Figure S5).

HCV was significantly associated with donor type. Replacement donors were 1.5 times more likely to be HCV sero-reactive compared to volunteer donors (OR = 1.50; 95% CI 1.03, 2.20). This might be probably because volunteer blood donors donate blood in a regular manner who know their sero-status for HCV infection before donating blood. Thus, volunteer blood donors were found to be relatively safer than replacement donors in which blood banks should follow stringent donor selection criteria with an emphasis on getting more volunteer blood donors. However, sex was not associated with HCV in this study.

Based on our review, the prevalence of syphilis was 1.50% (95% CI 1.20, 1.80%). This was almost consistent with the previous report from Kenya 1.0% [86]. On the other hand, a higher prevalence of syphilis was reported from Burkina Faso 3.96% [10] and Nigeria 4.2% [87]. In contrast to the current finding, a lower prevalence of syphilis was reported from earlier studies in Pakistan 0.43% [88] and India 0.43% [89]. The possible reason for the difference in the magnitude of syphilis across studies may be due to variation in risk behaviours in different geographical locations, or partly due to differences in socio-cultural practices.

On the other hand, male blood donors were more likely to be sero-reactive for syphilis (OR = 1.35; 95% CI 1.01, 1.79**)** compared to female donors. This might be due to differences in gender-related practices such as having multiple sexual partners. In addition, alcohol abuse is common among males that might expose males to high-risk behaviour which could attribute to an increased in the prevalence of syphilis. Furthermore, in some countries including Ethiopia, males make up the majority of blood donors. This could potentially be one factor as to why males had a higher odds ratio than females for TTIs. In this review, from the total 45 studies included in the review, 36 reported donor distribution by sex, and males accounted for 76.83% (324,590/422,499) of blood donors.

## Conclusion

In this systematic review, the pooled estimate of HBV, HIV, HCV and syphilis was high suggesting that there is a need to design and implement strategies to reduce the burden of TTIs in the general population. Moreover, creating community awareness about the prevention of TTIs should be strengthened. Moreover, being a replacement donor was also associated with high HBV, HIV and HCV infection. In addition, advanced and vigilance screening techniques of donated blood should be strengthened prior to transfusion. Furthermore, national policies and strategies of post-donation counselling for recruitment and retention of safe regular donors are a timely need. Lastly, conducting a national population-based survey for TTIs has of utmost importance to monitor the burden and trend of TTIs in the community.

The review has some limitations. One of the limitations is a high level of heterogeneity between included studies which can be attributed to differences in methodology, study period, and geographic location. Besides, there were differences in the diagnostic methods used across the studies which might be implicated with a high level of heterogeneity. Given these limitations, the review was conducted according to the preferred reporting items for systematic review and meta-analysis (PRISMA-P 2015 statement) protocol. Besides, a comprehensive searching of databases and the involvement of experts improved the quality of evidence generated.

## Supplementary Information

**Additional file 1: Table S1.** PRISMA 2009 checklist.

**Additional file 2: Figure S2.** A Plot of Egger’s test of publication bias for pooled estimate of HIV among Blood donors in Ethiopia.

**Additional file 3: Figure S3.** A Plot of Egger’s test of publication bias for pooled estimate of HBV among Blood donors in Ethiopia.

**Additional file 4: Figure S4.** A Plot of Egger’s test of publication bias for pooled estimate of HCV among Blood donors in Ethiopia.

**Additional file 5: Figure S5.** A Plot of Egger’s test of publication bias for pooled estimate of Syphilis among Blood donors in Ethiopia.

**Additional file 6: Figure S6.** Effect size of sex on HCV infection among blood donors in Ethiopia—a sub-group analysis by geographic region.

## Data Availability

The datasets used and/or analysed during the current study are available from the corresponding author on reasonable request.

## Abbreviations

FMoH: Federal Ministry of Health
HBV: Hepatitis B virus
HBsAg: Hepatitis B surface antigen
HBsAb: Hepatitis B surface antibody
HCV: Hepatitis C virus
HIV: Human immunodeficiency virus
OR: Odds ratio
TTI: Transfusion transmitted infection
WHO: World Health Organization
