# Supplementary material for: Sero-epidemiology and associated factors of HIV, HBV, HCV and syphilis among blood donors in Ethiopia: a systematic review and meta-analysis
Source: BMC Infect Dis. 2021 Aug 9;21:778. doi: 10.1186/s12879-021-06505-w (PMC8351159; doi:10.1186/s12879-021-06505-w)
Supplement: Supplementary file 4 — Additional file 4: Figure S4. A Plot of Egger’s test of publication bias for pooled estimate of HCV among Blood donors in Ethiopia. [file 12879_2021_6505_MOESM4_ESM.doc]

**Additional file 4: figure S4**. A Plot of Egger’s test of publication bias for pooled estimate of HCV among blood donors in Ethiopia
