# Supplementary material for: Sero-epidemiology and associated factors of HIV, HBV, HCV and syphilis among blood donors in Ethiopia: a systematic review and meta-analysis
Source: BMC Infect Dis. 2021 Aug 9;21:778. doi: 10.1186/s12879-021-06505-w (PMC8351159; doi:10.1186/s12879-021-06505-w)
Supplement: Supplementary file 6 — Additional file 6: Figure S6. Effect size of sex on HCV infection among blood donors in Ethiopia—a sub-group analysis by geographic region. [file 12879_2021_6505_MOESM6_ESM.doc]

**Additional file 6: Figure 56**: Effect size of sex on HCV infection among blood donors in Ethiopia – a sub-group analysis by geographic region
